# The Microhardness and Surface Roughness Assessment of Bulk-Fill Resin Composites Treated with and without the Application of an Oxygen-Inhibited Layer and a Polishing System: An In Vitro Study

**DOI:** 10.3390/polym14153053

**Published:** 2022-07-28

**Authors:** Ann Carrillo-Marcos, Giuliany Salazar-Correa, Leonor Castro-Ramirez, Marysela Ladera-Castañeda, Carlos López-Gurreonero, Hernán Cachay-Criado, Ana Aliaga-Mariñas, Alberto Cornejo-Pinto, Luis Cervantes-Ganoza, César Félix Cayo-Rojas

**Affiliations:** 1 School of Stomatology, Universidad Privada San Juan Bautista, 15066 Lima, Peru; ann.carrillo@upsjb.edu.pe (A.C.-M.); giuliany.salazar@upsjb.edu.pe (G.S.-C.); leonor.castro@upsjb.edu.pe (L.C.-R.); hernan.cachay@upsjb.edu.pe (H.C.-C.); acornejop@unfv.edu.pe (A.C.-P.); 2 “Grupo de Investigación Salud y Bienestar Global”, Faculty of Dentistry and Postgraduate School, Universidad Nacional Federico Villarreal, 15001 Lima, Peru; mladera@unfv.edu.pe (M.L.-C.); aaliagam@unfv.edu.pe (A.A.-M.); 3 School of Stomatology, Universidad Científica del Sur, 15067 Lima, Peru; clopezg@cientifica.edu.pe; 4 Faculty of Stomatology, Universidad Inca Garcilaso de la Vega, 15084 Lima, Peru; luiscervantesganoza@outlook.com

**Keywords:** bulk-fill resin, comparative study, dental materials, dental polishing, dentistry, oxygen-inhibited layer, resin composite, surface roughness, surface microhardness

## Abstract

The aim of this study was to assess the microhardness and surface roughness of bulk-fill resin composites treated with and without the application of an oxygen-inhibited layer (OIL) and a polishing system. This in vitro experimental study consisted of 72 resin composite blocks divided into three groups: Tetric N-Ceram Bulk Fill, Opus Bulk Fill APS, and Filtek Bulk Fill. Each resin composite group was further divided into two subgroups: with and without OIL control. Subsequently, surface roughness and microhardness were measured before and after polishing. A *t*-test was used to compare independent and related measures. For the intergroup comparison of variation before and after polishing, the Kruskal–Wallis test with Bonferroni post hoc was used considering a significance level of *p* < 0.05. When comparing surface roughness, significant differences were observed between Opus Bulk Fill resin composite with and without OIL control (*p* = 0.003) before polishing. The same occurred when comparing Tetric N-Ceram resin composite with and without OIL control (*p* = 0.039) after polishing. In addition, the surface roughness of Filtek Bulk Fill, Opus Bulk Fill, and Tetric N-Ceram Bulk Fill resin composites, with and without OIL control, decreased significantly after polishing (*p* < 0.001), while surface microhardness significantly increased (*p* < 0.05), with the exception of Opus Bulk Fill resin with OIL control (*p* = 0.413). In conclusion, OIL control and polishing significantly improved the surface roughness and surface microhardness of Filtek Bulk Fill and Tetric N-Ceram Bulk Fill resin composites. However, in the case of Opus Bulk Fill resin composite, only its surface roughness was significantly improved.

## 1. Introduction

Currently, resin composites continue to be the most widely used restorative materials due to their excellent esthetics, functional capacity, and mechanical properties [1,2,3]. With the evolution of these materials, bulk-fill resin composites appeared, which allow a monoblock technique to be used, placing a restoration with a 4–5 mm thick layer and light-curing easily [1,2,3,4], as they are more translucent and have less filler. Likewise, as the filler particles have a lower refractive index [5], they can replace both enamel and dentin [3], reducing operative times, shrinkage during polymerization, and air entrapment between the layers generated when using conventional resin composite and incremental technique [1,2].

The composition of bulk-fill composite resins is similar to that of conventional ones. However, each manufacturer adds some modifications to improve their properties such as modified monomers, flexible fillers, or even photoinitiators to achieve correct polymerization and reduce polymerization stress [6].

Bulk-fill resin composites have become a good product of choice due to their quality in terms of strength and durability, presenting high biocompatibility and better physical properties, such as greater wear resistance and surface hardness, as they are formed by nanoparticles and ceramic metal fillers that improve the resin surface, thus facilitating modeling and polishing with a better esthetic finish [7]. These characteristics have made its appearance successful, since it facilitates the reduction in clinical working time in a class I cavity by allowing a maximum incremental thickness of 4 mm to be light-cured with limited contraction, making it possible to fill the cavity in a single step [1,2]. Likewise, since they have a good adaptive capacity, the interproximal wall in a class II cavity can be first restored to transform it into a class I cavity, thus reducing the possibility of harmful effects on marginal integrity [8]. In addition, they are a good alternative for non-cooperative patients [2]. However, restorations based on resin composites can be affected by the formation of rough surfaces that can cause staining, plaque accumulation, gingival irritation, recurrent caries, and wear kinetics, among other problems [9,10,11].

Studies [12,13,14] have revealed the importance of resin composite reaction to polymerization in the presence of atmospheric oxygen, as this can affect the surface layer of the resin composite by producing free radicals that can bind to the Bis-GMA monomer and oxygen itself. These oxygen-free radical bonds are characterized by the formation of a stable peroxide radical and are non-reactive. The stable free radical bonding results in the non-polymerization of monomer residues on the resin composite surface. The unpolymerized remnants on the surface constitute what is called an oxygen-inhibited layer [12]. This layer contains the residual monomers which, due to decreased conversion, obtain less hardness on the resin composite surface [13]. Its thickness varies from 2.5 to 50 µm, in visible light-cured resin composites [14]. Due to this, the use of glycerin is recommended before light-curing the last layer of resin composite, as it forms a physical barrier that optimizes the conditions of light-curing processes by acting as an inhibitor of atmospheric oxygen that converts highly reactive radicals into relatively stable hydroperoxides, allowing a better curing quality in the outermost layer of composite resins [15,16].

On the other hand, a technique widely accepted by the dental community to preserve the mechanical properties of resin composite surfaces is the polishing and finishing system, which is based on considerably reducing surface roughness, since its presence reduces durability and produces bacterial plaque accumulation, color variation, and loss of brightness [17]. For this reason, finishing and polishing procedures are of great importance in dental restoration processes, since they reduce rough surfaces and, at the same time, attenuate the formation of the oxygen-inhibited layer, achieving less pigmented surfaces with ideal aesthetics that last over time [18]. In addition, resin composites containing nanoparticles are less susceptible to particle detachment through contact with abrasive material from polishing systems, favoring the reduction in surface roughness [13].

The superficial microhardness of resin composites is important for the clinical success of restoration, since the higher the microhardness of restorative material, the better the resistance to surface wear and scratching [10,11]. Therefore, it is important to improve this mechanical property on the surface by subjecting it to polishing procedures, eliminating rough surfaces that would eventually affect the resin composites’ resistance to chewing forces, since small surface reliefs can fracture and facilitate the retention of bacterial plaque and even facilitate the formation of secondary caries [12,13,14].

Surface roughness, as a consequence of irregularities in the application of restorative materials, is a clinical problem, making it necessary to perform some finishing and polishing techniques to avoid later stains, plaque presence, recurrent deterioration, etc. [19,20]. Surface texture is a critical point of vital importance to ensure the longevity of the restoration. Therefore, the use of multiple fine and superfine diamond rotary cutting instruments, aluminum oxide abrasive discs such as coarse-to-fine grain discs, as well as soft rubber discs impregnated with diamond and silicone, is recommended [21,22,23].

Studies such as those by Babina et al. [24], Madhyastha et al. [20], and St-Pierre et al. [25] have reported similar limitations such as the operator variable and the type of movement performed during polishing, so they recommend that the whole procedure should be performed by a single operator. In addition, Aljamhan et al. [26] and Khudhur et al. [27] mentioned that to assess surface properties such as roughness or others, it is advisable to make an initial measurement for better comparison; all the above-mentioned factors were taken into account to prepare the present study.

More studies are needed regarding the surface properties of bulk-fill resin composites due to the scarcity of scientific studies in the literature on this topic. Most of the studies related to oxygen-inhibited layer focus on bond strength testing [28]. Therefore, the aim of the present study was to assess the microhardness and surface roughness of bulk-fill resin composites treated with and without the application of an oxygen-inhibited layer and a polishing system. The null hypothesis was that (I) there are no significant differences in the microhardness and surface roughness of bulk-fill resins treated with and without the application of an oxygen-inhibited layer, and (II) there are no significant differences after polishing system procedures.

## 2. Materials and Methods

### 2.1. Type of Study and Delimitation

This in vitro experimental, longitudinal, and prospective study was conducted at the Stomatology School of the Universidad Privada San Juan Bautista and at the High Technology Certified Laboratory (ISO/IEC Standard: 17025), Lima, Peru, from January to March 2022, with approval letter No.1583-2021-CIEI-UPSJB. The CRIS Guidelines (Checklist for Reporting In Vitro Studies) were considered in the present study [29].

### 2.2. Sample Calculation and Selection

**Figure 1:**
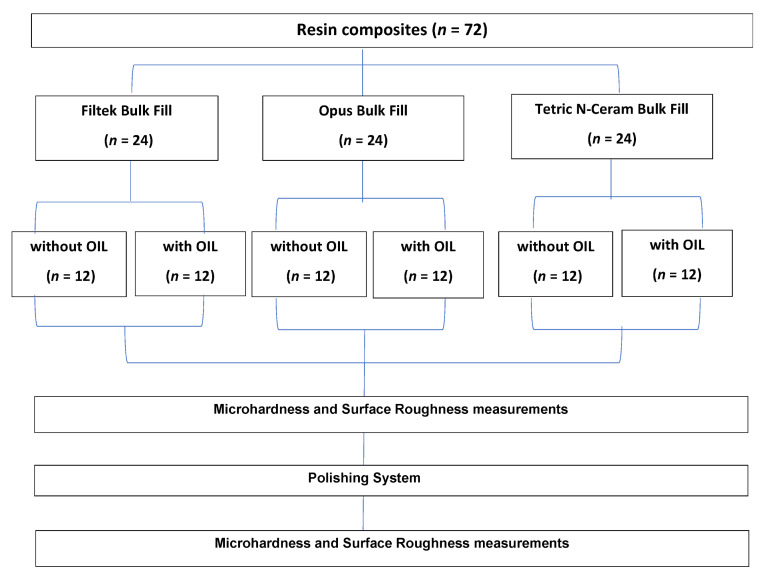
Random distribution of groups according to resin composite type, glycerin use, and polishing type.

A total of 72 resin composite blocks were made and standardized and evenly distributed in three groups of 24 blocks. These were divided in a simple random fashion without replacement into two equal subgroups of resin composite blocks with glycerin (*n* = 12) and without glycerin (*n* = 12) (Figure 1). The total sample size (*n* = 72) was calculated based on the data obtained in a previous pilot study in which the formula for analysis of variance was applied in G*Power statistical software version 3.1.9.7 considering a significance level (α) = 0.05 and statistical power (1 − β) = 0.80, with an effect size 0.39 with 6 groups and 2 paired measures. The data for sample size calculation considered microhardness and surface roughness, and based on these, the highest sample size was chosen.

### 2.3. Sample Characteristics and Sample Preparation

**Figure 2:**
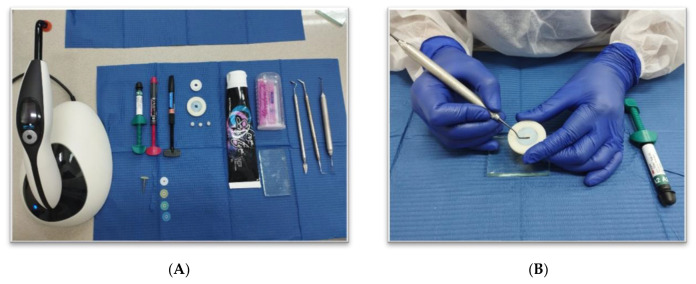
(**A**) Materials and instruments used; (**B**) resin composite compaction inside the block.

**Figure 3:**
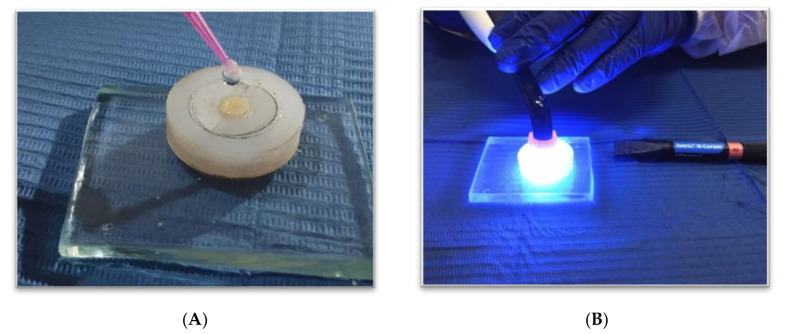
(**A**) Application of glycerin prior to light-curing; (**B**) light-curing of resin composite.

For the present study, the units of analysis were 72 bulk-fill resin blocks (Table 1), made by a single operator measuring 6 mm in diameter and 4 mm in depth [17]. The resin groups were coded and distributed as follows (Table 1 and Figure 2):

For groups without glycerin application and without polishing, a 1 mm thick microscope slide was used, making sure that the upper and lower surfaces were parallel. The resin composite samples were light-cured from the top of the mold with a light-emitting diode (LED) (Bluephase^®^, Ivoclar© Vivadent, Schaan, Liechtenstein) curing lamp with an intensity of 1200 mW/cm^2^ for 20 s [3,4,6]. The intensity was verified by a radiometer (Bluephase^®^ Meter II Dental Radiometer, Ivoclar© Vivadent, Schaan, Liechtenstein). For groups with glycerin DeOxTM (Ultradent, South Jordan, UT, USA) application and no polishing, the same procedure was followed, only before light-curing the last increment, a layer of glycerin was applied to the sample surface and light-cured from the top of the mold at the same intensity and time (Figure 3).

### 2.4. Microhardness and Surface Roughness Testing

All 72 resin composite blocks were measured for microhardness and surface roughness prior to the polishing procedure. After that, the sample was stored in an oven at 37 °C for 24 h. Then, the same operator polished all the resin composite block surfaces with an electric motor (EM-E6, W&H, Bürmoos, Austria) and a contra-angle handpiece (NSK, Tokyo, Japan) for 20 s per step according to the manufacturer’s specification with a four-step coarse-to-superfine grain disc system (Sof-Lex, 3M ESPE, St. Paul, SM, USA) at speed of 15,000 rpm with identical movements and in the same direction. Then, microhardness and surface roughness were measured again, followed by washing and drying the samples to remove surface residues.

**Figure 4:**
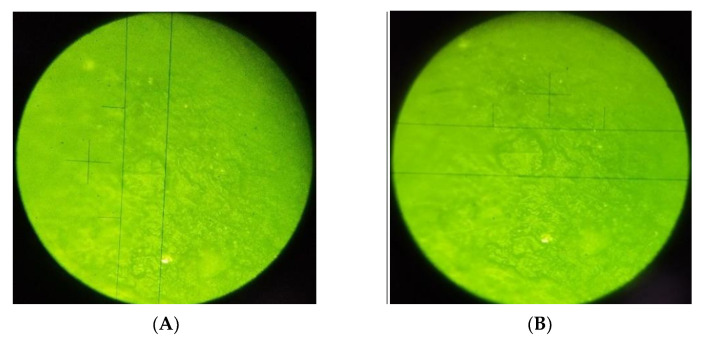
(**A**) Opus Bulk Fill without OIL control; (**B**) Opus Bulk Fill with OIL control.

**Figure 5:**
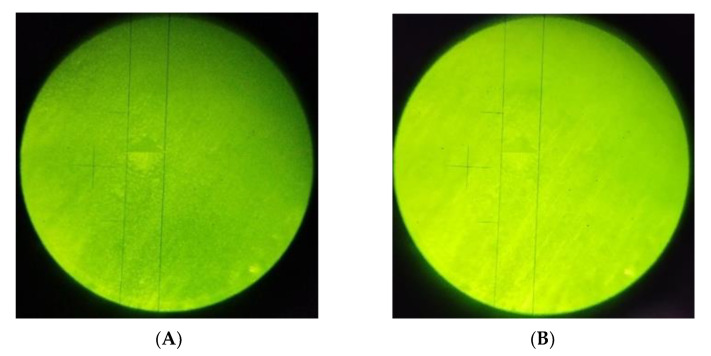
(**A**) Tetric N-Ceram Bulk Fill without OIL control; (**B**) Tetric N-Ceram Bulk Fill with OIL control.

**Figure 6:**
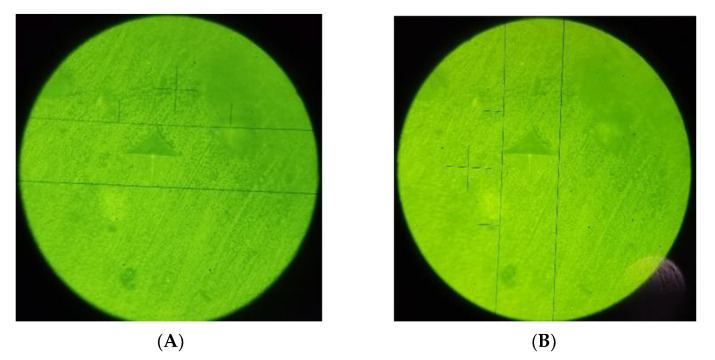
(**A**) Filtek Bulk Fill without OIL control; (**B**) Filtek Bulk Fill with OIL control.

Surface microhardness was measured with an Electronic Vickers microhardness tester (HVS-1000 Jinan Liangong Testing Technology Co., Ltd., Shandong, China) with a 1-micron approximation at 40×. Four notches were made in the middle of the resin composite block surface, under a 100 g-f load for 10 s at different points with the same distance between them and maintaining a minimum distance of 1 mm adjacent to the sample’s margins. The surface microhardness value (kg/mm^2^ = HV (Vickers hardness)) was determined by dividing the load applied to the indentation surface (Figure 4, Figure 5 and Figure 6).

Surface roughness was determined as the average of absolute roughness (Ra) in microns of four measurements taken on the other half of the resin composite block surface using a digital roughness meter with a resolution of 0.001 microns (SRT-6200^®^, Huatec, Beijing, China).

### 2.5. Statistical Analysis

The collected data were recorded in a Microsoft Excel 2019^®^ file and subsequently imported for statistical analysis using SPSS (*Statistical Package for the Social Sciences Inc*. IBM, Armonk, NY, USA) version 28.0. For descriptive analysis, measures of central tendency and dispersion such as mean and standard deviation were used. For hypothesis testing, the Shapiro–Wilk test and Levene’s test were used to evaluate whether the data presented normal distribution and homoscedasticity, respectively. According to the results, in the difference of means, normal distribution was observed in all groups (before and after polishing), so it was decided to use the *t*-test for independent and related measures. However, for intergroup comparison of the variation between before and after polishing, the nonparametric Kruskal–Wallis test with Dunnet’s post hoc and Bonferroni correction was used. A significance level of 5% (*p* < 0.05) was considered for all comparisons.

## 3. Results

Before polishing, Filtek Bulk Fill (2.42 ± 0.86 µm), Opus Bulk Fill (3.10 ± 1.34 µm), and Tetric N-Ceram Bulk Fill (3.48 ± 1.54 µm) resin composites with OIL control presented higher surface roughness. However, after polishing, the Filtek Bulk Fill resin composite with OIL control presented higher surface roughness values (0.61 ± 0.22 µm) than the same without OIL control (0.50 ± 0.21 µm), while Opus Bulk Fill presented similar surface roughness values with OIL control (0.52 ± 0.33 µm) and without OIL control (0.52 ± 0.19 µm). In addition, the Tetric N-Ceram without OIL control presented higher surface roughness values (0.79 ± 0.48 µm) than the same with OIL (0.45 ± 0.21 µm) (Table 2). After the *t*-test, it could also be seen that all the resin composites, with and without oxygen-inhibited layer control, decreased their surface roughness after polishing. Finally, all the surface roughness values of the analyzed resin composites showed normal distribution (*p* > 0.05) (Table 2).

Before polishing, the resin composites with the highest surface microhardness were the Filtek Bulk Fill without OIL control (45.67 ± 1.87 HV) and Opus Bulk Fill (45.88 ± 3.90 HV) and Tetric N-Ceram Bulk Fill (44.43 ± 3.49 HV) both with OIL control. On the other hand, after polishing, it could be observed that the Filtek Bulk Fill with and without OIL control (48.22 ± 3.78 HV and 49.68 ± 1.98 HV, respectively) and the Tetric N-Ceram Bulk Fill with and without OIL control (47.32 ± 1.93 HV and 46.99 ± 2.80 HV, respectively) presented similar surface microhardness values. However, Opus Bulk Fill presented higher surface microhardness values with OIL (46.50 ± 3.37 HV) than the same without OIL (38.70 ± 6.19 HV) (Table 3). All the resin composites with and without oxygen-inhibited layer control increased their surface microhardness after polishing. Finally, all the surface microhardness values of the analyzed resin composites presented normal distribution (*p* > 0.05) (Table 3).

When comparing surface roughness, significant differences were observed between the Opus Bulk Fill resins with and without OIL control (*p* = 0.003) before polishing. In addition, significant differences were observed when comparing the Tetric N-Ceram resin with and without OIL control (*p* = 0.039), after polishing. (Table 4).

Significant differences were also observed before polishing when comparing the surface microhardness with and without the Filtek Bulk Fill, Opus Bulk Fill, and Tetric N-Ceram Bulk Fill resin control (*p* = 0.018, *p* < 0.001, and *p* = 0.007, respectively). In addition, after polishing, only significant differences were observed when comparing the Opus Bulk Fill resin with and without OIL control (*p* = 0.001) (Table 4).

The surface roughness of Filtek Bulk Fill, Opus Bulk Fill, and Tetric N-Ceram Bulk Fill resin composites, all with and without OIL control, decreased significantly (*p* < 0.001) after polishing. However, their surface microhardness with and without OIL control significantly increased (*p* < 0.05) after polishing, with the exception of the Opus Bulk Fill with OIL control, which showed no significant difference after polishing (*p* = 0.413) (Table 5).

When comparing the surface roughness variation before and after polishing between bulk-fill resin composites with and without OIL control, significant differences were observed (*p* = 0.001). The Tetric N-Ceram Bulk Fill with OIL control showed significantly greater variation than Filtek Bulk Fill (*p* = 0.038) and Opus Bulk Fill (*p* = 0.006) both without OIL control. In addition, the Opus Bulk Fill with OIL control showed significantly greater variation than the same without OIL control (*p* = 0.019) (Table 6).

When comparing the surface microhardness variation before and after polishing between bulk-fill resin composites with and without OIL control, significant differences were observed (*p* = 0.001). The Opus Bulk Fill with OIL control showed significantly less variation than the Filtek Bulk Fill with OIL control (*p* = 0.013) and Tetric N-Ceram Bulk Fill without OIL control (*p* = 0.001) (Table 6).

## 4. Discussion

The surface properties of resin composites, roughness, and microhardness have gained great clinical importance, as they are related to the esthetics and function of restorations. The absence of these properties results in periodontal disease and the development of secondary caries due to increased plaque accumulation and wear of the restoration, compromising long-term clinical success [30]. Therefore, any restorative material should reproduce the biological, functional, and esthetic properties of a natural tooth. With the evolution of restorative materials, bulk-fill resin composites emerged offering improved physical and mechanical properties that depend on their composition, which varies according to manufacturers, as they can modify the organic matrix, size, and morphology of the filler particles to achieve adequate behavior [5]. However, oxygen in contact with the resin composite can influence the polymerization reaction by forming the OIL, thus compromising the surface properties of this restorative material [1,31,32]. Currently, there is still no consensus in the dental community as to whether glycerin and/or the polishing and finishing system should be applied independently or in a complementary manner to optimally preserve the mechanical properties on the resin composite surface when it comes into contact with atmospheric oxygen at the time of the final light-curing [10,28]. The aim of the present study was to assess the microhardness and surface roughness of bulk-fill resin composites light-cured with and without the application of an oxygen-inhibited layer and a polishing system. The null hypothesis was rejected since the resin composites with and without oxygen-inhibited layer control decreased their surface roughness and increased their surface microhardness after polishing, in agreement with the results obtained by Suares et al. [33] and Zhang L. et al. [34]. Gantz et al. [35] reported surfaces with lower roughness only in groups with OIL, possibly because they used 70% alcohol for 20 s to control OIL, which has been recommended by previous studies such as Tupinamba et al. [36] and Panchal et al. [31]. In the present study, glycerin was used because, in addition to controlling OIL, it improves the conversion degree of resin composites, obtaining better surface properties and allowing to achieve a smooth surface with the absence of porosity and microcracking [37,38].

A Sof-Lex disc was used in the present study for 20 s to polish the surface of resin composites following the manufacturer’s instructions, as it has been reported that this allows obtaining a lower surface roughness compared with any other polishing system [35,39,40]. It should be noted that the surface roughness of all resin composites decreased significantly after polishing, with and without OIL control, with final values ranging from 0.0025 µm to 0.8 µm, which is acceptable according to the ISO 1302:2002 quality standard [41].

Among the resin composites used, after polishing, the Tetric N-Ceram without OIL control presented higher roughness values, which could be due to the presence of OIL, which would affect its conversion degree. In addition, this resin composite had a lower filler content than the others, as well as pre-polymerized modified fillers and an elastic filler, which, taken together, could decrease Young’s modulus of elasticity, generating greater deformation of the surface [6]. Possibly, by presenting greater flexibility, the surface could be affected by heat due to the friction generated by the Sof-Lex discs, causing microcracks in the matrix polymer and, consequently, a rougher surface [38,42,43]. However, controlling the oxygen-inhibited layer with glycerin would prevent the contact of the resin composite with atmospheric oxygen and improve its degree of conversion and surface properties [9,12,27], thereby reducing the surface roughness.

The surface microhardness of resin composites has been defined as the resistance to indentation or abrasion [42,44]. There is also a relationship between the filler characteristics (size, weight, volume) and the chemical composition of the resin composites [41,44]. Thus, the chemical composition and filler content in the matrix of resin composites affect their physical properties such as surface microhardness [43]. For this reason, it is claimed that materials with high filler content would have higher surface hardness since, immediately after curing, the surface layer, mainly composed of the organic matrix, can further polymerize during polishing, thus increasing its strength [45,46]. Therefore, to ensure a successful restoration, resin composites should have a surface hardness as close as possible to the surface of natural teeth [44,47,48].

The surface microhardness test results, both before and after polishing, revealed that the Opus Bulk Fill resin composite showed significant differences when comparing the OIL control versus the non-control. These findings could be due to the fact that the Opus Bulk Fill resin composite resin works with a new *advanced polymerization system* (APS) technology that reduces the amount of camphorquinone by incorporating other types of initiators and co-initiators that are secrets of the brand and do not require activation with light in the violet spectrum, amplifying the polymerization capacity and increasing the conversion degree and depth of LED curing, which would improve the mechanical and surface properties [49]. It should be noted that the matrix composition is not well-described by the manufacturer [50]. Therefore, according to what was obtained in the present study, we can assume that this APS technology present in Opus Bulk Fill was improved by the OIL control, allowing it to function properly as described by the manufacturer, resulting in better surface microhardness. Regarding surface roughness, it was found that this resin composite with and without OIL control reduced its values significantly after applying the polishing system, so it was deduced that polishing the resin composite can provide a smoother finish [4,12,15], improving surface roughness. However, when controlling the OIL of the Opus Bulk Fill resin composite, there were no significant changes in surface microhardness between before and after polishing, unlike the significantly higher changes in Filtek Bulk Fill and Tetric N-Ceram resin composites. This is probably because Tetric N-Ceram Bulk Fill contains alternative photoinitiators intended to enhance photopolymerization, such as ivocerin (a dibenzoyl germanium derivative) and monoacylphosphine oxide (TPO), which are stimulated by different wavelengths [2,3,49]. Previous studies have shown that ivocerin acts as a polymerization enhancer, allowing it to be efficient [2,3,49]. Filtek Bulk Fill is a composite resin that has camphorquinone as a photoinitiator, with an absorption peak of approximately 470 nm that matches the wavelength emitted by most LED-curing lights on the market [51], which could improve the conversion degree of the resin composite by increasing its surface microhardness, as it has been shown with a variety of different composite resins that 80% of maximum hardness is associated with 90% of maximum polymerization [52,53,54].

In the present study, the decision to control the oxygen-inhibited layer with glycerin and not with celluloid matrix is due to the fact that Lassila et al. [55] and Strnad et al. [56] suggested that, although celluloid tape does control OIL by blocking the contact of the material with oxygen, it could trap bubbles during placement, which could affect polymerization on the surface. Furthermore, according to Soliman et al. [57] and Park et al. [58], a celluloid tape is not applicable in a real clinical scenario and could only be used for some interproximal surfaces but not for occlusal surfaces due to the presence of elevations and depressions. The application of glycerin would be more effective in accessing all surfaces and controlling OIL formation by converting the highly reactive surface radicals into relatively stable hydroperoxides, allowing better surface quality, avoiding contact with atmospheric oxygen, and thus creating the conditions to improve the degree of conversion and surface properties [15,18,32]. On the other hand, polishing with Sof-Lex discs has limitations when used on the occlusal surfaces of posterior teeth because the grooves and elevations do not allow a complete surface approach, preventing the complete removal of OIL. Therefore, their use is recommended in areas with smooth surfaces such as the buccal surfaces of anterior teeth [59].

The results of the present study suggest an alternative to control the oxygen-inhibited layer, taking into account that it can influence the surface properties of resin composites. Likewise, these results allow us to recommend the use of glycerin in combination with a polishing system to counteract the formation of this layer. This could contribute to improving the survival rate of restorations since it has been reported that having poor surface properties can lead to pigment retention, plaque, the possibility of fracture, and secondary caries formation [9,10,11,30]. As a strength of the present study, it should be mentioned that several authors such as Wheeler et al. [12], Paravina et al. [21], Espindola et al. [51], and Bouschlicher et al. [53] have assessed only one surface property of resin composites, while in the present study, two surface properties could be assessed in the same sample unit, reducing the bias that would be obtained by assessing surface microhardness and surface roughness in different study units. Furthermore, according to the obtained results, it is evident that the Tetric N-Ceram Bulk Fill and Filtek Bulk Fill resin composites with OIL control and after polishing improved their surface properties, which could favor a better esthetic and functional performance under masticatory forces [25,39,40,46], while in the case of Opus Bulk Fill, only its surface roughness was significantly improved, which could allow a better esthetic performance but not necessarily an improvement in its resistance to masticatory forces. It should be noted that the Opus Bulk Fill resin composite, before being polished with OIL control, already had similar surface properties to Filtek Bulk Fill and Tetric N-Ceram Bulk Fill, but these properties were affected by the lack of OIL control in this resin composite.

Among the limitations of the present study, it is recognized that since it is an in vitro investigation, the obtained results could not be extrapolated to the clinical field; for this reason, it would be advisable to develop randomized clinical trials with the same proposed aim. It is recognized that the present study procedure may be different from a clinical situation, since changes in temperature, the presence of saliva, enzymes, and changes in pH could affect microhardness and surface roughness over time. In addition, within the polishing methodology of resin composites, it is recognized that the digital pressure variable could not be controlled. However, as a strength of the study design, it was possible to control the rpm, polishing direction, and prevention of crack formation due to water cooling.

It is recommended for future studies to assess these properties in clinical situations with thermal cycling or other tests that simulate clinical conditions, in addition to polishing in the presence of water, as recommended by some authors such as Gönülol et al. [60], who reported that this would be favorable since, in addition to extracting the heat, water filters the eroded particles that should be immediately removed from the surface of the restoration. It is also recommended to assess the relationship that could exist between the conversion degree of resin composites used in the present study with their photoinitiators.

## 5. Conclusions

Within the limitations of the present in vitro study, it can be concluded that polishing and control of the oxygen-inhibited layer significantly improved the surface roughness and microhardness of Filtek Bulk Fill and Tetric N-Ceram Bulk Fill resin composites. However, in the case of the Opus Bulk Fill resin composite, it only presented significant improvements with respect to its surface roughness.

## Figures and Tables

**Table 1:** Technical profile of products used.

Product	Type	Composition	Filler % (wt—vol)	Manufacturer	Lot
Filtek™ Bulk Fill A2 (F-BF)	Nanofill Bulk Fill	Matrix: AUDMA, UDMA, AFM y 1, 12-dodecane-DMA Filler: not agglomerated/not aggregated silica, not agglomerated/not aggregated zirconia, aggregated zirconia/silica compound, ytterbium trifluoride	76.5 wt% 58.4 vol%	3M, ESPE, St. Paul, MN, USA	NE24741
Tetric^®^ N-Ceram Bulk Fill IVA (TNC-BF)	Nanohybrid Bulk Fill	Matrix: bis-GMA, bis-EMA, UDMA Filler: barium silicate alumino glass, “isofiller” (prepolymer, glass, and ytterbium fluoride), ytterbium fluoride, and mixed oxides	76 wt% 54 vol%	Ivoclar Vivadent, Schaan, Liechtenstein	Z02GG2
Opus Bulk Fill APS A2 (O-BF)	Nanohybrid Bulk Fill	Matrix: UDMA Filler: Nanofiller Photoinitiation -Advanced Polymerization System (APS). Inorganic load of silanized silicon dioxide (silica), barium glass aluminosilicate	76.5 wt% 58.4 vol%	FGM, Santa Catarina, Brazil	010221/191021
Sof-Lex System	Finishing Polishing system	Aluminum oxide abrasive discs	SL Coarse: 60 μm SL Medium: 29 μm SL Fine: 14 μm SL Superfine: 5 μm	3M, ESPE, St. Paul, MN, USA	46817

**Table 2:** Analysis of surface roughness values (µm) before and after polishing of bulk-fill resin composites with and without OIL control.

Resin Composite	Glycerin	Polishing	*n*	Surface Roughness (µm)							
				Mean	SD	SE	95% CI		Min	Max	* *p*
							LL	UL			
**F-BF**	Yes	Before	12	2.42	0.86	0.25	1.87	2.96	1.26	3.88	0.592
	No		12	1.78	0.81	0.23	1.27	2.30	0.88	3.59	0.146
**O-BF**	Yes		12	3.10	1.34	0.39	2.25	3.95	0.41	5.20	0.847
	No		12	1.58	0.65	0.19	1.16	1.99	0.38	2.61	0.897
**TNC-BF**	Yes		12	3.48	1.54	0.44	2.50	4.45	1.26	5.82	0.579
	No		12	2.51	0.86	0.25	1.96	3.06	1.55	4.36	0.128
**F-BF**	Yes	After	12	0.61	0.22	0.06	0.47	0.75	0.25	0.88	0.165
	No		12	0.50	0.21	0.06	0.37	0.64	0.03	0.82	0.720
**O-BF**	Yes		12	0.52	0.33	0.10	0.31	0.73	0.07	1.05	0.218
	No		12	0.52	0.19	0.05	0.40	0.64	0.18	0.80	0.913
**TNC-BF**	Yes		12	0.45	0.21	0.06	0.32	0.58	0.20	0.78	0.296
	No		12	0.79	0.48	0.14	0.49	1.10	0.16	1.57	0.337

*n*: sample size; F-BF: Filtek Bulk Fill, O-BF: Opus Bulk Fill, TNC-BF: Tetric N-Ceram Bulk Fill; SD: standard deviation; SE: standard error of the mean; 95% CI: 95% confidence interval, LL: lower limit, UL: upper limit; * based on Shapiro–Wilk normality test, *p* > 0.05 (normal distribution). OIL: oxygen-inhibited layer.

**Table 3:** Analysis of surface microhardness (HV) values, before and after polishing, of bulk-fill resin composites with and without OIL control.

Resin Composite	Glycerin	Polishing	*n*	Surface Microhardness (HV)							
				Mean	SD	SE	95% CI		Min	Max	* p
							LL	UL			
**F-BF**	Yes	Before	12	40.82	5.92	1.71	37.06	44.58	31.00	50.30	0.688
	No		12	45.67	1.87	0.54	44.48	46.86	42.80	48.50	0.713
**O-BF**	Yes		12	45.88	3.90	1.13	43.40	48.35	39.70	51.80	0.872
	No		12	32.26	4.68	1.35	29.29	35.23	25.40	38.50	0.269
**TNC-BF**	Yes		12	44.43	3.49	1.01	42.21	46.64	38.80	49.40	0.601
	No		12	38.74	5.43	1.57	35.29	42.19	29.70	45.20	0.154
**F-BF**	Yes	After	12	48.22	3.78	1.09	45.81	50.62	42.80	54.20	0.474
	No		12	49.68	1.98	0.57	48.42	50.94	46.90	52.90	0.502
O-BF	Yes		12	46.50	3.37	0.97	44.36	48.64	41.20	51.00	0.114
	No		12	38.70	6.19	1.79	34.77	42.63	27.60	47.80	0.929
**TNC-BF**	Yes		12	47.32	1.93	0.56	46.09	48.54	43.80	50.40	0.898
	No		12	46.99	2.80	0.81	45.21	48.77	42.20	52.30	0.962

*n*: sample size; F-BF: Filtek Bulk Fill, O-BF: Opus Bulk Fill, TNC-BF: Tetric N-Ceram Bulk Fill; SD: standard deviation; SE: standard error of mean; 95% CI: 95% confidence interval, LI: lower limit, UL: upper limit; * based on Shapiro–Wilk normality test, *p* > 0.05 (normal distribution).

**Table 4:** Comparison of OIL control effect on surface roughness (µm) and microhardness (HV) of bulk-fill resin composites before and after polishing.

Resin Composite	Polishing	Glycerin	*n*	Surface Roughness (µm)			Surface Microhardness (HV)		
				Mean	SD	* *p*	Mean	SD	* *p*
**F-BF**	Before	Yes	12	2.42	0.86	0.077	40.82	5.92	0.018
		No	12	1.78	0.81		45.67	1.87	
	After	Yes	12	0.61	0.22	0.242	48.22	3.78	0.251
		No	12	0.50	0.21		49.68	1.98	
**O-BF**	Before	Yes	12	3.10	1.34	0.003	45.88	3.90	<0.001
		No	12	1.58	0.65		32.26	4.68	
	After	Yes	12	0.52	0.33	0.994	46.50	3.37	0.001
		No	12	0.52	0.19		38.70	6.19	
**TNC-BF**	Before	Yes	12	3.48	1.54	0.074	44.43	3.49	0.007
		No	12	2.51	0.86		38.74	5.43	
	After	Yes	12	0.45	0.21	0.039	47.32	1.93	0.744
		No	12	0.79	0.48		46.99	2.80	

F-BF: Filtek Bulk Fill, O-BF: Opus Bulk Fill, TNC-BF: Tetric N-Ceram Bulk Fill; SD: standard deviation; * based on Student’s *t* for independent measures, *p* < 0.05 (significant differences).

**Table 5:** Comparison of surface roughness (µm) and microhardness (HV) between before and after polishing of bulk-fill resin composites with and without OIL control.

Resin Composite	Glycerin	Test	Difference (Ⴟf − Ⴟi)	SD	SE	95% CI		*t*	* *p*
						LL	UL		
**F-BF**	Yes	SR	−1.81	0.88	0.25	−2.37	−1.25	−7.10	<0.001
		SM	7.40	5.91	1.70	3.65	11.15	4.34	0.001
	No	SR	−1.28	0.91	0.26	−1.86	−0.71	−4.89	<0.001
		SM	4.02	2.63	0.76	2.34	5.69	5.28	<0.001
**O-BF**	Yes	SR	−2.58	1.24	0.36	−3.37	−1.79	−7.22	<0.001
		SM	0.63	2.55	0.73	−0.99	2.24	0.85	0.413
	No	SR	−1.06	0.63	0.18	−1.45	−0.66	−5.86	<0.001
		SM	6.44	6.81	1.97	2.11	10.77	3.28	0.007
**TNC-BF**	Yes	SR	−3.03	1.55	0.45	−4.01	−2.04	−6.74	<0.001
		SM	2.89	2.03	0.59	1.60	4.18	4.93	<0.001
	No	SR	−1.72	0.92	0.26	−2.30	−1.14	−6.50	<0.001
		SM	8.25	4.99	1.44	5.08	11.42	5.72	<0.001

F-BF: Filtek Bulk Fill, O-BF: Opus Bulk Fill, TNC-BF: Tetric N-Ceram Bulk Fill; SR: surface roughness; SM: surface microhardness; Ⴟf: mean after polishing; Ⴟi: mean before polishing; SD: standard deviation; SE: standard error of mean; 95% CI: 95% confidence interval, LL: lower limit, UL: upper limit; * based on Student’s *t* for related measures, *p* < 0. 05 (significant differences).

**Table 6:** Comparison of microhardness (HV) and surface roughness (µm) variation, before and after polishing, between bulk-fill resin composites with and without OIL control.

Test	Resin Composite	Average (Ⴟf − Ⴟi)	Median	IQR	Z	* *p*
**SR**	F-BF (G)	−1.8095 ^a,c^	−1.4830	1.20	19.96	0.001
	F-BF	−1.2835 ^a,b^	−1.2180	1.13		
	O-BF (G)	−2.5803 ^a,b^	−2.5640	1.74		
	O-BF	−1.0573 ^c^	−1.1375	1.02		
	TNC-BF (G)	−3.0263 ^b^	−2.8065	2.92		
	TNC-BF	−1.7181 ^a,b,c^	−1.6485	0.87		
**SM**	F-BF (G)	7.4000 ^a,c^	6.2500	11.08	20.36	0.001
	F-BF	4.0167 ^a,b^	3.8000	3.38		
	O-BF (G)	0.6250 ^b^	0.4500	2.58		
	O-BF	6.4417 ^a,b,c^	8.1000	12.90		
	TNC-BF (G)	2.8917 ^a,b,c^	2.9500	3.03		
	TNC-BF	8.2500 ^a,c^	6.9000	6.18		

SR: surface roughness; SM: surface microhardness; F-BF: Filtek Bulk Fill, O-BF: Opus Bulk Fill, TNC-BF: Tetric N-Ceram Bulk Fill; G: with glycerin; Ⴟf: mean after polishing; Ⴟi: mean before polishing; IQR: interquartile range; Z: Kruskal–Wallis test: * *p* < 0.05 (significant differences). ^a,b,c^: different letters indicate significant differences (*p* < 0.05) based on Bonferroni post hoc.

## Data Availability

The data presented in this study are available on request from the corresponding author.
